# Diagnostic Value of Bronchoalveolar Lavage in Leukemic and Bone Marrow Transplant Patients: The Impact of Antimicrobial Therapy

**DOI:** 10.4084/MJHID.2015.002

**Published:** 2015-01-01

**Authors:** Abraham Tareq Yacoub, Dani Thomas, Carol Yuan, Carolina Collazo, John Greene, Frank Walsh, David Solomon, Skai Schwartz, Arthur Andrews

**Affiliations:** 1 Moffitt Cancer Center, 12902 Magnolia Drive. Tampa, Florida 33612-9497; 2 University of South Florida. Morsani College of Medicine. Moffitt Cancer Center; 3 University of South Florida; 4 James A. Haley Veterans Administration Hospital. University of South Florida. Morsani College of Medicine. Moffitt Cancer Center

## Abstract

There is significant morbidity and mortality from pneumonia in leukemic and bone marrow transplant patients. We sought to explore the diagnostic yield of bronchoalveolar lavage (BAL) in these patients with new pulmonary infiltrates. A retrospective chart review of approximately 200 Non- human immunodeficiency virus (HIV) leukemic and Hematopoietic stem cell transplantation (HSCT) patients who underwent bronchoscopy at a single academic cancer center was performed. Antimicrobial use for less than 24 hours at the time of BAL was associated with a higher yield in this population (56.8% versus 32.8%, p<0.001). This supports performing bronchoscopy with BAL within 24 hours of antimicrobial therapy in leukemic and HSCT patients.

## Introduction

Pneumonia carries significant morbidity and mortality in leukemic and bone marrow transplant patients.^1^,^2^ The development of pulmonary infiltrates in the setting of such immunocompromise raises concern for both infectious and non-infectious etiologies, some of which are potentially treatable. Many of these patients are receiving broad-spectrum antimicrobials when they develop the infiltrates, either for prophylactic or treatment purposes. The use of bronchoscopy as a diagnostic tool in these immunocompromised patients with lung infiltrates has been well described and is common in clinical practice.^3^,^4^ Performing bronchoscopy provides several different options for sampling the lower respiratory tract. Among these, bronchoalveolar lavage (BAL) is especially effective at collecting samples from the alveoli and has been shown to be associated with less risk than transbonchial biopsy.^4^,^5^ Indeed, BAL has been documented as a diagnostic tool for identifying causative pathogens as well as non infectious etiologies in immunocompromised populations^3^ and is common clinical practice. The reported diagnostic yield of BAL in immunocompromised patients, including those with HIV or solid organ transplant, with pulmonary infiltrates ranges widely from 22 to 80%.^5^–^9^ Studies of BAL sensitivity in hematopoietic stem cell transplant (HSCT) patients report yields of 22–65%.^6^,^7^,^9^–^12^ With respect to the yield of BAL in non-resolving pneumonia one study in the general intensive care unit (ICU) setting, Pereira Gomes reported a 72% yield in 53 patients.^13^ We sought to examine the effect of antimicrobial treatment on BAL results in a large study population of leukemic and bone marrow transplant patients.

## Subjects and Methods

This retrospective chart review was performed at a single academic cancer center. A power analysis was performed to determine the appropriate sample size. Estimating from the literature specific to our population^6^,^7^,^9^,^11^,^12^,^14^–^16^ we calculated a sample size of 300 with a power of 0.8 to detect an increase in BAL yield of 23% if the BAL was obtained within 24 hours of antibiotic use. The patients were selected from those who had undergone an inpatient bronchoscopy in reverse chronological order until 300 patients with either a hematopoietic stem cell transplant or hematologic malignancy were identified. The exclusion criteria were age <18, a diagnosis of HIV or acquired immune deficiency syndrome (AIDS), or outpatient status. Electronic medical records were reviewed and data extracted by a single investigator, CY. Data including age, sex, cancer diagnosis, time from HSCT, leukocyte count, neutropenia in addition to medications were collected. A normal white blood cell (WBC) count was considered 4,000–12,000/mm^3^. Neutropenia was defined as an absolute neutrophil count (ANC) less than 500/mm^3^. Medications including antibiotic duration and timing, antifungal use, immunosuppressant use or glucocorticoids were recorded. Glucocorticoids were converted to prednisone equivalents and were documented if the patient had received at least 20mg daily for > 2 months or 60mg daily for > 3 weeks. A positive BAL yield was defined as the culture identification of at least one organism known to be pathogenic in this patient population. Candida species and coagulase negative *staphylocci* were considered colonizers. Our infectious disease expert, JG, clarified discrepancies. The bronchoscopy technique and procedure was similar for each patient, utilizing a Fujinon 470S bronchoscope for every procedure, with the same systematic methodology, as is the routine at this academic cancer center. Each bronchoscopy was performed by an attending physician or by a pulmonary fellow with direct attending supervision. The BAL specimens were collected without suction connected to the bronchoscope prior to a systematic airway survey. The BAL was performed by instilling two 60cc aliquots of room temperature sterile 0.9% saline followed by slow manual aspiration. The specific subsegmental bronchus from which the BAL specimens were obtained was recorded. In addition, the volume yield and color of each specimen were documented in most cases.

Correlates of a positive BAL yield and time on antibiotics were initially analyzed via a chi-square test, or a Fisher’s exact test if the expected count was less than 5. Assuming a binomial distribution, a log-risk model was employed to estimate the risk ratio of a positive yield with respect to antibiotic duration less than 24 hours. An adjusted risk ratio was estimated by adjusting this model for the factors found to be associated with a positive BAL yield and time on antibiotics. Statistical analyses were performed with Statistical Analysis Software Version 9.3.

## Results

A total of 302 patient records were evaluated. Three patient records were lacking BAL data. Two patient records were lacking data about antimicrobial therapy. These patients were therefore excluded from antibiotic related statistical analyses, leaving a total of 297 patients. All but 5 patients were on antifungal therapy. One patient had insufficient data about antibiotic duration, and one lacked adequate medication data.

The age range was 18–85 with an average age of 53.5. Thirty eight percent of the patients were female and 41.8% of the patients had undergone HSCT. A minority of the patients required mechanical ventilation either at the time of BAL or within 48 hours. These demographic data are presented in Table 1.

One hundred seven of the 297 patients had a positive BAL culture for an overall BAL yield of 36%. There was not a statistically significant difference between the overall incidence of a positive BAL yield and antibiotic duration. There was a significant association when the patients were stratified according to a 24-hour timeframe. Of the 37 patients on antibiotics for less than 24 hours, including all 4 patients who were not on any antibiotics, twenty-one (56.8%) had a positive BAL culture compared with eighty-five of the 259 (32.8%) patients who had been on antibiotics longer than 24 hours at the time the BAL specimen was obtained and had a positive culture, (p<0.001). Among patients who were on antibiotics for 24 to 48 hours or >48 hours at the time of BAL, the yields were essentially the same at 30.8% and 30.3%, respectively. Among the patients who were on antibiotics for more than 48 hours, 118 of them had their antibiotic regimen changed within 48 hours of their BAL. The BAL culture was positive in 42 (35.6%) of these patients. There were 27/89 (30.3%) patients on antibiotics for more than 48 hours who did not have their regimen changed within 48 hours of their positive BAL. These data are presented in Table 2.

Forty-eight patients were not receiving chemotherapy or immunosupressants, 15 (31.3%) of whom had a positive BAL yield. One hundred twenty-six patients were on chemotherapy and 32 (25.4%) had a positive BAL culture. Forty-one patients were not on chemotherapy but were on immunosupressants and 14 (34.1%) had a positive BAL yield. Eighty patients were on both chemotherapy and immunosupressants and 46 (57.5%) had a positive BAL culture. This is illustrated in Table 3.

We evaluated leukocyte count and neutropenia with respect to BAL yield. There were 64 patients with a normal WBC count and 30 (46.9%) had a positive BAL culture. Patients with an abnormal WBC or frank neutropenia were less likely to have a positive BAL yield with 39.8% and 27.7% positive yield, respectively.

Whether the patient was on a ventilator was also evaluated. There were 23 patients on mechanical ventilation at the time of their BAL and 12 (52.2%) had positive cultures. 90 of 269 (33.5%) patients who were not mechanically ventilated had a positive BAL. This is depicted in Table 3.

Prior to dichotomizing the duration of antibiotics, the association of antibiotic duration with a positive BAL yield was not statistically significant. Table 4 compares patients who were on antibiotics for at least 24 hours prior to obtaining the BAL specimen to those who were on antimicrobials less than 24 hours before the procedure. Patients who were on antibiotics for at least 24 hours were significantly less likely to have a positive BAL yield (32.8% vs. 56.8%, p<0.01). They were also more likely to have leukemia, an abnormal WBC count, and less likely to be on a combination of chemotherapy and immunosupressants together. The risk ratio for a positive yield adjusted for leukemia, abnormal WBC count, and concurrent chemotherapy with immunosuppressant agents was 0.734 (95%CI 0.519–1.037, p=0.078).

There were no statistically significant differences in the BAL effluent color, volume, or whether it originated from the right or left lung with respect to incidence of positive BAL yield.

Among the positive yields, there was no significant difference in overall distribution of pathogen type. There was a non-significant trend toward a lower incidence of fungi in those on antibiotics for at least 24 hours. (Tables 5, 6 and 7)

## Discussion

This study evaluated the diagnostic utility of BAL in a sizeable number of leukemic and HSCT patients with pulmonary infiltrates who were on antimicrobials. The overall yield of a positive BAL result in our study, 36.0%, was within the wide range documented in other studies of HSCT populations. The duration of antimicrobial therapy was related to the diagnostic yield of BAL within a 24 hour timeframe. Patients who were on antibiotics for less than 24 hours at the time of their BAL were more likely to have a pathogen identified. This correlates with Shannon’s findings of an improved BAL sensitivity in early versus late BAL in a similar population.^10^ Interestingly, among patients already on antimicrobial therapy for >48h at the time of BAL, a change in the regimen during the 48h preceding BAL was associated with a better yield. This may be a reflection of sample size, a new infection, inappropriate antimicrobial change, severity of illness, or less likely colonization. To our knowledge, this finding has not discretely been reported in this patient population. Whether or not this positive result represents true infection is unknown based on the available data from this study. Pereira Gomes reported a 72% yield in critically ill patients with unresolving pneumonia, and over 90% of these patients were on antibiotics at the time of BAL.^13^ Our findings are in contradiction to those reported by Souweine who examined ventilator associated pneumonia in a general ICU population and found a BAL sensitivity of 71% among patients not on antibiotics 4 days prior to bronchoscopy, 83% in patients on antibiotics >72h at the time of BAL, and 38% in patients with new antibiotics within 24 hours of BAL.^17^ It is important to note that a minority of our patients were supported with mechanical ventilation and that our study did not examine ventilator-associated pneumonia (VAP) in a general ICU population. In our study neutropenic patients were less likely to have a positive BAL result. This was similar to the finding reported by Shannon who had a 32% yield in patients with an ANC <100 and a 50% yield in patients with an ANC <500.^10^ Mechanically ventilated patients had a higher BAL yield. This may be a reflection of the small sample size or severity of illness. The overall BAL yield was higher in patients with acute leukemia, mechanical ventilatory support, lack of neutropenia, and a combination of chemotherapy and other immunosuppressant agents. It is common clinical practice to perform BAL in leukemic and bone marrow transplant patients with unexplained new lung infiltrates. This study supports the practice of obtaining a BAL specimen within 24 hours of antimicrobial therapy in leukemic and HSCT patients with unexplained new lung infiltrates, a population that is universally on antimicrobials at the time of BAL.

It is important to acknowledge several limitations of this study. Among them is its retrospective design, the lack of a comparison control group, and selection bias as the included patients underwent pulmonary consultation at the discretion of the primary service. The single center nature of the study with regional antimicrobial prophylaxis and treatment practices as well as local pathogen resistance patterns may impair the ability to apply the findings elsewhere. In addition, the study could be improved by attempting to correlate BAL yield with radiographic imaging patterns, more clinical parameters such as fever, hypoxemia, level of acuity, any available anatomic or cytological specimens, and comparison with temporal noninvasive microbiologic analyses. Designing the study to attribute a diagnosis of infectious or non-infectious etiology of the lung infiltrates would be helpful as this population is prone to pulmonary infiltrates from drug toxicities, alveolar hemorrhage, malignancy, radiation, pulmonary edema, graft versus host disease (GVHD), and bronchiolitis obliterans among others. Assessing treatment changes and mortality outcomes with the timing of bronchoscopy will be of value in future study.

## Figures and Tables

**Table 1:** 

Patient Characteristic	Number
Female	113 (38.0%)
HSCT	124 (41.8%)
Acute Leukemia	194 (65.3%)
Mechanical Ventilation	28 (9.4%)
**Time on antibiotics**	
None	4 (1.35%)
<24 hours	33 (11.1%)
24–48 hours	52 (17.6%)
>48 hours, without change	89 (30.1%)
>48 hours, with change	118 (39.9%)
**WBC**	
Normal	64 (21.5%)
< 4000 or > 12000	103 (35.7%)
Neutropenia	130 (43.8%)
**Medication**	
Chemotherapy	126 (42.7%)
Immunosupressants	41 (13.9%)
Combined	80 (27.1%)
Neither	48 (16.3%)

**Table 2:** Stratification of patients by time on antibiotics and the likelihood of a positive bronchoalveolar lavage (BAL) result. (NS = not significant)

Time on antibiotics (hrs)	Positive BAL/number patients (%)	p value
None	3 / 4 (75.0)	NS
<24 hours	18/33 (54.5)	
24–48 hours	16/52 (30.8)	
>48 hours w/o change	27/89 (30.3)	
>48h, but with change	42/118 (35.6)	
0–24 hours	21/37 (56.8)	<0.01
> 24 hours	85/259 (32.8)	

**Table 3:** BAL yield with respect to ventilator status, WBC, and pharmacotherapy.

Characteristic	Number of patients (%)	Positive BAL Number of patients (%)
**Ventilation**		
Mechanical Ventilation	28 (9.4%)	12 (52.2%)
No Mechanical Ventilation	269 (90.6%)	90 (33.5%)
**WBC**		
Normal	64 (21.5%)	30 (46.9%)
< 4000 or > 12000	103 (35.7%)	41 (39.8%)
Neutropenia	130 (43.8%)	36 (27.7%)
**Medication**		
Chemotherapy	126 (42.7%)	32 (25.4%)
Immunosupressants	41 (13.9%)	14 (34.1%)
Combined	80 (27.1%)	46 (57.5%)
Neither	48 (16.3%)	15 (31.3%)

BAL indicates Bronchoalveolar lavage; WBC, white blood cell.

**Table 4:** Patient characteristics stratified by time on antibiotics at the time of BAL sampling.

Characteristic	Time on antibiotics <24 hours Number (%)	Time on antibiotics ≥ 24 hours Number (%)	P Value
Sample Size	37 ( 100)	259 ( 100)	
**Age Group**			< 0.01
21–49	10 (27.0)	77 (29.7)	
50–69	26 (70.3)	119 (45.9)	
70+	1 (2.70)	63 (24.3)	
% Female	15 (40.5)	97 (37.5)	NS
BAL Yield, % with pathogenic growth	21 (56.8)	85 (32.8)	< 0.01
%Leukemia	18 (48.6)	176 (68.0)	<0.05
**Ventilator**			NS
not vented	35 (94.6)	233 (90.0)	
On vent	2 (5.41)	21 (8.11)	
vented <48h after BAL	0 ( 0 )	5 (1.93)	
**WBC**			<0.001
normal	18 (48.6)	45 (17.4)	
<4000 or >12000	14 (37.8)	89 (34.4)	
neutropenic	5 (13.5)	125 (48.3)	
**Medication**			<0.001
Missing data	1 (2.70)	1 (0.39)	
None	8 (21.6)	40 (15.4)	
Chemo	2 (5.41)	124 (47.9)	
Immunosupressants	6 (16.2)	35 (13.5)	
Combined	20 (54.1)	59 (22.8)	
**BAL Return volume**			NS
Missing data	1 (2.70)	5 (1.93)	
<30cc	1 (2.70)	14 (5.41)	
30–59cc	21 (56.8)	125 (48.3)	
60–89cc	13 (35.1)	98 (37.8)	
90–120cc	1 (2.70)	17 (6.56)	
**BAL Lobe**			NS
Missing data	1 (2.70)	22 (8.49)	
Right	24 (64.9)	162 (62.5)	
Left	12 (32.4)	75 (29.0)	

**Table 5:** Distribution of pathogens detected with respect to antibiotic duration.

	Time on antibiotics <24 hours Number (%)	Time on antibiotics ≥24 hours Number (%)
**Bacteria Only**	5 (23.8)	30 (35.3)
**Bacteria and Fungi only**	3 (14.3)	14 (16.5)
**Bacteria and Virus only**	1 (4.76)	5 (5.88)
**All three pathogens**	3 (14.3)	3 (3.53)
**Fungi/yeast only**	5 (23.8)	9 (10.6)
**Fungi and Virus only**	1 (4.76)	5 (5.88)
**Virus Only**	3 (14.3)	19 (22.4)

**Table 6:** Incidence of organisms detected with respect to antibiotic duration. (NS = not significant)

	Time on antibiotics <24 hours	Time on antibiotics ≥24 hours	P Value
**Bacteria (Total)**	12/21 (57.1)	52/85 (61.2)	NS
**Fungi/yeast (Total)**	12/21 (57.1)	31/85 (36.5)	0.05 < p <0.1
**Virus (Total)**	8/21 (38.1)	32/85 (37.6)	NS
**All three**	3/21 (14.3)	3/85 (3.50)	0.05 < p <0.1
**Two or more**	8/21 (38.1)	27/85 (31.8)	NS

**Table 7:** Pathogens isolated in the cultures.

Virus	Bacteria	Fungi

Cytomegalovirus	*Achromobacter*	*Arthroconidia*
	*Acinetobacter*	*Arthrographis*
Respiratory syncytial virus	*Acinetobacter baumannii*	*Aspergillus flavus*
	*Alcaligenes Xylosoxidans*	*Aspergillus fumigatus*
Herpes simplex virus		*Aspergillus terreus*
	*Bipolaris*	*Aspergillus versicolor*
Influenza A virus		
Influenza B virus	*Enterobacter cloacae*	*Candida guilliermondii*
	*Enterococcus faecium*	*Candida inconspicua*
Parainfluenza virus	*Enterococcus Gallinarum*	*Candida glabrata*
	*Escherichia Coli*	*Candida krusei*
		*Candida parapsilosis*
	Group F *Streptococcus*	*Candida tropocalis*
		*Cladosporium*
	*Haemophilus parainfluenza*	*Cunninghamella*
	*Klebsiella pneumoniae*	*Fusarium*
	*Lactobacillus*	*Malbranchea species*
	*Legionella pneumophila*	*Mucor circinelloides*
		*Penicillium*
	Methicillin-resistant *Staphylococcus aureus*	*Pneumocystis jiroveci pneumonia*
	*Moraxella catarrhalis*	
	*Mycobacterium Avium-Intracellulare*	*Ramichloridium Schulzeri*
	*Mycobacterium abscessus*	
	*Mycobacterium gadium*	*Saccharomyces cerevisiae*
	*Mycobacterium mucogenicum*	*Scedosporium apiospermum*
	*Mycobacterium phocaicum*	*Scytalidium*
	*Nocardia*	*Candida parapsilosis*
	*Pseudomonas aeruginosa*	
	*Pseudomonas mendocina*	
	*Serratia marcescens*	
	*Stenotrophomonas Maltophilia*	
	*Streptococcus pneumoniae*	
